# Subgroup identification of disparities in buprenorphine discontinuation in opioid-use disorder: A Virtual Twins machine learning approach using nationwide United States claims data, 2006–2022

**DOI:** 10.1371/journal.pmen.0000469

**Published:** 2026-04-09

**Authors:** Kehe Zhang, Paula A. Jaimes-Buitron, Wanru Guo, Yanmin Gong, Yuanxiong Guo, Carolina Vivas Valencia, Cici Bauer

**Affiliations:** 1 Department of Biostatistics and Data Science, School of Public Health, The University of Texas Health Science Center at Houston, Houston, Texas, United States of America; 2 Center for Spatial-Temporal Modeling for Applications in Population Sciences, School of Public Health, The University of Texas Health Science Center at Houston, Houston, Texas, United States of America; 3 Department of Biomedical Engineering and Chemical Engineering, The University of Texas at San Antonio, San Antonio, Texas, United States of America; 4 School of Engineering Medicine, Texas A&M University, Houston, Texas, United States of America; 5 Department of Information Systems and Cybersecurity, The University of Texas at San Antonio, San Antonio, Texas, United States of America; Northern Ontario School of Medicine: NOSM University, CANADA

## Abstract

Buprenorphine retention is crucial for effective treatment of opioid use disorder (OUD), yet disparities in treatment discontinuation persist. This study aims to identify and quantify disparities in buprenorphine treatment retention using a machine learning framework adapted from Virtual Twins approach focusing on disparities related to sex, age, insurance type, geographic region, mental health status and community-level Social Vulnerability Index (SVI). Using a nationwide longitudinal cohort, we applied a two-stage machine learning approach. In Stage one, we trained classification models to estimate the counterfactual differences in treatment discontinuation across disparity types. Model performance was assessed using C-statistics and precision-recall curve. In Stage two, we employed regression models, decision trees and neural networks with Shapley Additive Explanations, to identify subgroups most vulnerable to the disparities and key contributing factors. Among 303,528 treatment episodes from 131,169 patients aged 18–85, 71% discontinued treatment within 180 days. Early medication adherence (3-month proportion of days covered) was the strongest predictor. Significant disparities emerged based on insurance, region, mental health status, age, and SVI. Higher discontinuation risk was observed among privately insured older adults, patients from high-SVI areas in the South without mental health diagnoses, and younger publicly insured individuals lacking psychiatric services. Psychiatric service utilization consistently mitigated discontinuation risks across subgroups. Limitations include the absence of race/ethnicity in the claims data, the inability to capture concurrent medications and initial buprenorphine dose, and lack of formal uncertainty estimates for the quantified disparities. The Virtual Twins analytical framework enabled identification of vulnerable subgroups and quantification of disparities attributable to specific risk factors. Interventions that prioritize early adherence, expand access to psychiatric services, and address structural barriers in high-SVI Southern communities and insurance-defined risk groups may improve equity in buprenorphine retention.

## Introduction

Opioid use disorder (OUD) is a major public health crisis, affecting millions globally and imposing substantial social and economic burdens. In 2022, there were nearly 110,000 deaths attributed to drug overdose in the US, with over 81,000 involving opioids – a 400% increase over the past decade [1]. From 2013 to 2022, the age-adjusted overdose death rates from synthetic opioids other than methadone increased significantly from 1.0 to 22.7 per 100,000 population [2]. Effective treatment strategies are needed to mitigate these consequences [3]. In the United States, opioid agonist treatment is delivered within a highly regulated system in which methadone is dispensed only through federally certified opioid treatment programs and buprenorphine has historically been prescribed in office-based settings under separate regulatory requirements [4], in contrast to more integrated models such as Canada where both medications are available within routine care [5]. Buprenorphine, an FDA-approved medication for opioid use disorder (MOUD) in the U.S., is one of the most effective treatments [6]. Studies have demonstrated its efficacy in reducing opioid cravings, overdose risk, and mortality, particularly when maintained for at least six months [7].

The utilization of buprenorphine for at least 180 days is recognized as a benchmark of treatment quality, as extended durations are associated with improved outcomes [8–13]. In addition, prior studies have demonstrated that retention at one year is strongly associated with sustained clinical benefit, including reduced substance use, lower relapse risk, and improved psychosocial functioning [14,15]. However, the optimal length of treatment has not been empirically established. Despite its effectiveness, maintaining consistent treatment and ensuring patient retention continue to be significant challenges. In 2022, only approximately 25% of the estimated 4% of U.S. adults requiring OUD treatment received recommended medications [16]. Recent studies have shown substantial variability in buprenorphine retention rates, with many reporting rates below 50% across different populations and treatment settings [17–19]. For example, an analysis of 2011–2015 Health Care Cost Institute commercial claims data showed that over 55% of patients discontinued buprenorphine treatment within the first six months [19]. Similarly, a study examining an all-payer pharmacy claims database found that only 41% of individuals had treatment lasting for at least 6 months [18]. Additionally, in a retrospective cohort study utilizing multi-state claims data, 50.6% patients discontinued their treatments within 180 days [20]. Importantly, treatment discontinuation increases the risk of opioid overdose mortality, suicide, and all-cause mortality [21–23]. Therefore, identifying factors associated with treatment discontinuation is essential for improving treatment retention and optimizing the impact of OUD treatment programs.

Existing research has documented disparities in access to and retention in MOUD treatment at both the individual and community level. These disparities include socioeconomic factors [24–26], geographic variation [27,28], race/ethnicity [29], insurance status [18], gender [30], and mental health comorbidities [31]. Studies have also identified key risk and protective factors for treatment discontinuation, such as demographic characteristics (sex, region, insurance type) [32,33], medical comorbidities (HIV, Hepatitis C, chronic pain) [17], and health services utilization [34–36]. In addition to individual-level risk factors, contextual community factors, including provider access [37], urban versus rural settings [38–40] and social vulnerability [41,42], have also been shown to impact MOUD treatment.

Despite these findings, several important gaps exist. First, most studies only examine a limited set of risk factors and neglect critical healthcare utilization factors. For example, Faysal et al. developed an explainable machine learning model incorporating key demographic and clinical factors along with early treatment adherence, but failed to account for health services utilization factors such as outpatient treatment services, medication management, group psychotherapy, and counselling, which are known to impact retention [43]. Studies have also been constrained to specific insurance types, geographic regions, or treatment settings, making their findings difficult to generalize. For example, Chan et al. (2024) [44], Sweeney et al. (2022) [35], and Vakkalanka et al. (2022) [34] focused exclusively on commercially insured patients or specific treatment modalities, such as telehealth- or primary care-based settings. Similarly, studies that relied solely on surveys [29] or interviews [30] may introduce potential bias and limit generalizability.

Recently, machine learning methods have increasingly been applied in MOUD research to identify risk factors and predict treatment outcomes, as demonstrated by Faysal et al. (2024) [45], Hasan et al. (2021) [46], and Warren et al. (2022) [8]. The Virtual Twins framework, originally introduced by Foster et al. in randomized clinical trial settings, provides a novel machine learning-based approach for identifying heterogeneity in treatment responses across subgroups [47]. In this framework, individual-level counterfactual outcomes under alternative treatment or exposure conditions are first estimated, and the resulting differences in predicted outcomes are subsequently modeled to identify subgroups with differential treatment responses [48]. This approach has since been adapted to examine disparities in substance use disorder treatment, which enables identification of subgroups with heterogeneous treatment effects and the factors contributing to these disparities [49,50]. However, prior applications were constrained by limited data coverage in terms of population, temporality, and geography, and none were focused on MOUD retention.

To address these gaps, we conducted a nationwide, longitudinal analysis leveraging the IQVIA PharMetrics Plus for Academics Closed Health Plan Claims database (2006–2023). Our objective was to quantify disparities in buprenorphine treatment discontinuation across key dimensions (sex, age, insurance type, region of residence, mental health status, and community-level social vulnerability) and to identify the subgroups and risk factors driving these disparities using a two-stage Virtual Twins framework with a counterfactual design [47]. Leveraging a large national dataset and both individual and community level factors, this study proposes a novel approach to identifying disparities in OUD treatment from multiple lenses, to inform policies aimed at promoting equitable access and retention.

## Methods

### Ethical statement

The study was reviewed and approved by the UTHealth Committee for the Protection of Human Subjects (CPHS; HSC-SPH-23-1114), with a waiver of informed consent for use of de-identified secondary data and no direct participant contact. IQVIA longitudinal prescription claims data (2006–2022) were accessed within the UTHealth Center for Health Care Data’s secure, access-controlled environment under a data-use agreement. Data were initially extracted and accessed on March 27^th^, 2024. The analytic dataset was de-identified; investigators had no access to direct identifiers or re-identification keys at any time.

### Data sources

We used IQVIA PharMetrics Plus for Academics Closed Health Plan Claims, a nationwide longitudinal claims database capturing prescription records in the U.S. from January 2006 to April 2023, with a total of 90,327,620 records for 256,737 individual patients. Each prescription record contains patient demographics (sex, age), geographic locations (from latest enrollment record), prescription claims details (e.g., National Drug Code [NDC], procedure and diagnostic codes, days and quantity of supply), prescription information (drug name, generic name, dosage form, strengths), diagnosis information for coexisting conditions (ICD-9/ ICD-10 codes and diagnosis description), procedures performed (CPT/ HCPCS and procedure description), and patient enrollment information (health insurance type, months of coverage). Geographic location measures available in IQVIA include region of residence (Northeast, Midwest, South, West, based on U.S. Census definitions) and 3-digit ZIP codes, which represent the first three digits of the standard 5-digit ZIP code and capture broader geographic areas while preserving confidentiality. We refer to patient-level variables as time-invariant information (e.g., demographics and geographic location), while episode-level variables characterize each treatment episode (e.g., duration in days). Details of the variable definitions are in Table A in S1 Appendix. In addition, we incorporate area-level socioeconomic variables based on the patients’ 3-digit ZIP code. Specifically, we used the Social Vulnerability Index (SVI) developed by the U.S. Centers for Disease Control and Prevention (CDC) for year 2022 [51]. The CDC SVI ranks 16 socioeconomic and demographic variables from the American Community Survey conducted by U.S. Census Bureau, including measures of socioeconomic status, racial and ethnic minority status, household characteristics and housing type and transportation. The SVI is a score ranging from 0 and 1, with higher values indicating greater vulnerability. Since the CDC SVI is reported by census tracts, it was mapped to 3-digit ZIP Code Tabulated Areas using population-weighted aggregation. The resulting values were rescaled to a 0–1 range and classified into four vulnerability levels based on quartiles: Low (0 - < 0.25), Medium-Low (0.25 - < 0.5), Medium-High (0.5 - < 0.75), and High (0.75 - 1.0).

### Study design and analytical cohort

**Fig 1:**
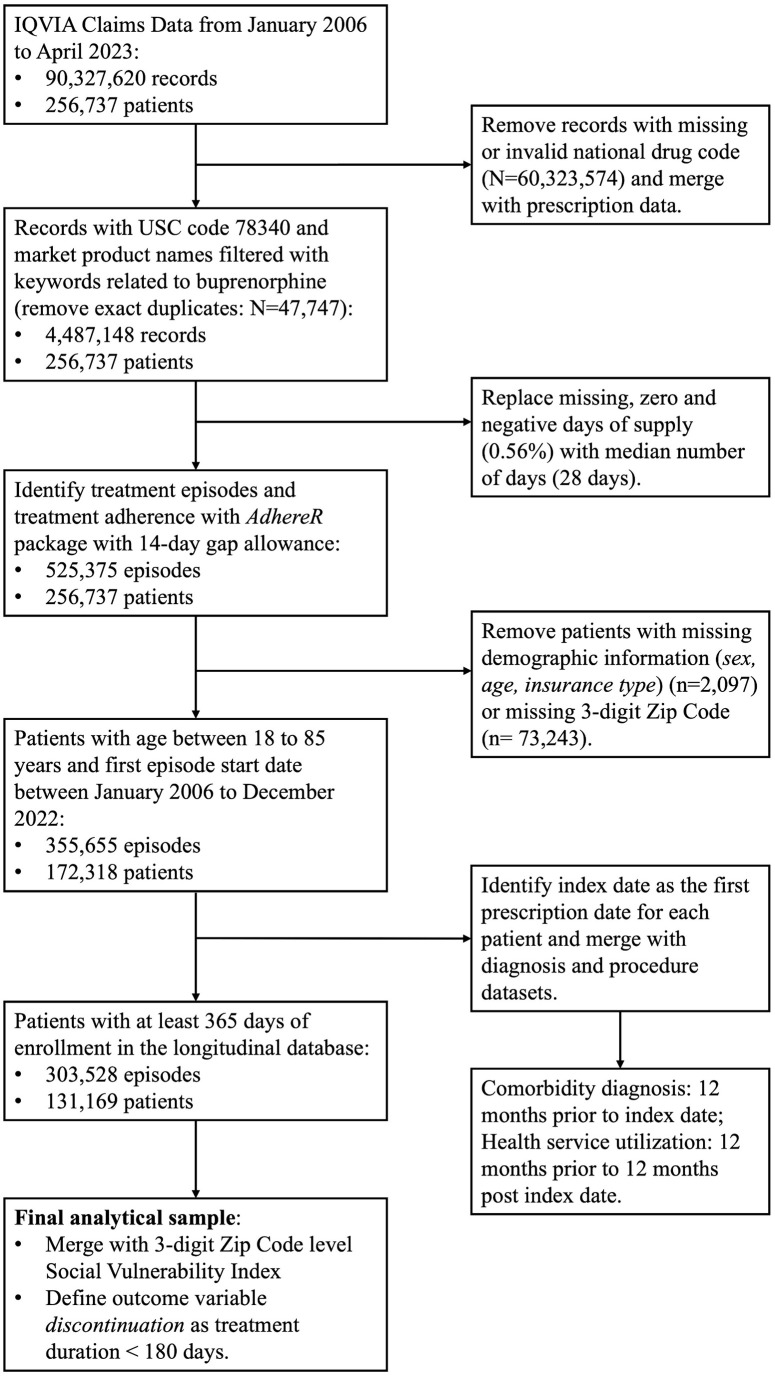
Flowchart of data processing and analytical cohort generation. N refers to the number of episodes and n refers to the number of patients.

This retrospective cohort study leveraged U.S. nationwide longitudinal claims data to construct buprenorphine treatment episodes and evaluate discontinuation among adult population. **Fig 1** summarizes the inclusion/exclusion criteria and procedures used to construct the analytical cohort. Buprenorphine claims for OUD were identified using the Uniform System of Classification (USC) code 78340 and refined by selecting prescriptions with buprenorphine-related product names and excluding exact duplicates. Buprenorphine treatment was defined to include all major formulations, including buprenorphine monoproducts, buprenorphine-naloxone combination products, and extended-release formulations (e.g., SUBLOCADE and PROBUPHINE), consistent with prior literature [52,53]. Sublingual products accounted for the vast majority of prescriptions (99.65%), whereas long-acting injectable formulations comprised a small proportion (0.35%). The initial screening yielded 4,487,146 records across 256,737 unique patients. Records with missing, zero, or negative values for “days of supply” (N = 24,947; 0.56%) were imputed using the population median, and extreme values were truncated at 90 days (N = 88; 0.002%), following the prior reports on prescribing practices [52,54,55]. Buprenorphine treatment episodes were constructed from prescription records, with a new episode defined as a prescription filled after a > 14-day gap in supply. This threshold was chosen based on a sensitivity analysis comparing alternative gaps of 7, 21, 30, and 60 days (Supplemental Results in S1 Appendix). For overlapping prescriptions, excess days were added to the end of the last prescription to calculate episode duration [52]. Episode construction was performed using the *AdhereR* package (version 0.8.1) in R [56,57]. We further excluded patients with missing demographic information (age, sex, and health insurance; N = 2,097) or invalid/missing 3-digit Zip Codes (N = 73,243). The primary outcome was buprenorphine treatment discontinuation, defined as a treatment episode lasting fewer than 180 days [52,58]. Episodes with a duration of <180 days were coded as discontinued (1), whereas episodes lasting ≥180 days were coded as continued (0).

Patient-level comorbidities were identified using ICD-10 and ICD-9 diagnostic codes present within the 12 months preceding the start date of the initial treatment episode, defined as the index date. Comorbidities conditions included alcohol use disorder, non-opioid substance use disorders, schizophrenia, bipolar disorder, anxiety disorder, posttraumatic stress disorder, depressive disorders, and HIV. Each comorbidity was coded as a binary indicator. Health service utilization was captured through CPT/HCPCS procedure codes occurring from 12 months before to 12 months after the index episode. These included outpatient medical services for general evaluations, outpatient psychiatric services, medication-assisted treatment counseling, telehealth services, induction and maintenance services, and in-clinic OUD services [59–61]. These variables captured the types of healthcare engagement that may influence adherence and treatment continuity. Detailed diagnostic and procedure codes are provided in Tables B and C in S1 Appendix.

To evaluate the early treatment adherence, we calculated the patient-level proportion of days covered (PDC), defined as the number of days covered by buprenorphine divided by the observation window [43]. PDC is a validated claims-based metric that quantifies the proportion of days a patient has medication available over a specified period [62,63]. PDC has been commonly used in prior studies of substance use disorder to assess early adherence and predict treatment retention and outcomes [43,46]. We compared 1-month and 3-month PDC measures and found that the 3-month PDC was more predictive of treatment discontinuation (Supplemental Results in S1 Appendix). Therefore, we used the 3-month PDC, defined as the number of days covered within the initial 90 days after treatment initiation divided by 90, in our final models. The final analytical cohort included 303,528 episodes from 131,169 patients aged between 18 and 85 who had at least one year of enrollment.

### Virtual twins machine learning model

**Fig 2:**
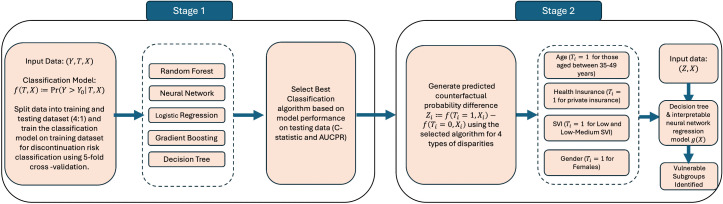
Two-stage Virtual Twins machine learning model diagram.

We employed a two-stage machine learning framework adapted from Virtual Twins approach to investigate the impact of disparities on treatment discontinuation risk. The modeling design and analytical workflow are illustrated in **Fig 2**. In Stage one, we trained a classification model to generate counterfactual predictions by toggling disparity variables (e.g., sex, age, health insurance) while holding other covariates constant, and computed the resulting individual-level disparity effects as differences in predicted discontinuation risk. In Stage two, we applied interpretable machine learning algorithms, including decision trees [64] and shallow neural network, to regress these disparity effects on patient characteristics, identifying subgroups most vulnerable to disparities. This two-stage approach separates disparity-effect estimation from subgroup identification and explanation, enhancing the interpretability of disparity-driven risk stratification. The coding and categorization of disparity variables used as predictors and as binary outcomes are summarized in Table A in S1 Appendix.

Let ${\left\{\left(Y_{i},\;T_{i},\;X_{i}\right)\right\}}_{i\;\in \;\left\{\text{1},\;\text{2},\;\ldots ,\;n\right\}}$ represent the treatment episode dataset, where $Y_{i}$ is the outcome variable - treatment discontinuation, $T_{i}$ is the disparity variable of interest (e.g., $T_{i}=\text{1}$ for females and $T_{i}=\text{0}$ for males), and $X_{i}$ is a vector of all other covariates for the i-th episode. In the first stage, we trained predictive models to estimate the probability of treatment discontinuation given the disparity type and all other covariates $f\left(T_{i},\;X_{i}\right)=\text{Pr}\left({Y_{i}=\text{1}\;|\;T}_{i}=\text{1},\;X_{i}\right).$ We considered different machine learning algorithms, including logistic regression, decision trees, random forest, gradient boosted machine (GBM) and neural network models. Three nested models were developed: Model 1 included baseline characteristics (sex, age, insurance type, geographic region, comorbidities, healthcare utilization); Model 2 added the SVI; and Model 3 further included the 3-month PDC variable. A complete list of variables included in each model is presented in Table D in S1 Appendix.

We randomly split the dataset into 80% training and 20% testing. Within the training data, five-fold cross-validation and grid search were used for model tuning. To address class imbalance, Synthetic Minority Oversampling Technique (SMOTE) was applied within training dataset. The optimal model was selected based on the highest C-statistic, defined as the area under the receiver-operating characteristic curve (AUC-ROC). Model performance was further evaluated using accuracy, precision, recall, and the area under the precision-recall curve (AUC-PR). For each observation, we computed a virtual difference $Z_{i}=f\left(T_{i}=\text{1},\;X_{i}\right)-f\left(T_{i}=\text{0},\;X_{i}\right)$, which captures the counterfactual probability change had the individual belonged to the alternative group (e.g., female vs. male) when all other covariates remained the same. We calculated the probability differences for sex (females vs. males), age group (35–49 vs. 18–34 and 50–85 combined), health insurance type (private vs. public insurance), SVI (low and low-medium vs. medium-high and high), region of residence (West vs. other regions), and mental health status (Yes vs. No). Mental health diagnosis refers to any diagnosis related to anxiety, depression, bipolar disorder, PTSD, and schizophrenia. Details on grid search for parameter tuning are included in Supplemental Methods in S1 Appendix.

In the second stage, the estimated virtual probability difference $Z_{i}$ from Stage one was treated as the primary outcome, and we fit regression models $g\left(X_{i}\right)$ adjusting all other covariates $X_{i}$, excluding the disparity variable. The covariates included in the model are healthcare services utilization, alcohol disorder, and non-opioid drug use 12 months prior to the index date. We considered interpretable machine learning methods such as decision trees and shallow neural network models to identify key factors contributing to disparities. Decision trees were used to define subgroups most vulnerable to discontinuation disparities, while neural networks were paired with SHAP (Shapley Additive Explanations) [65] values to quantify global and local feature importance. SHAP is a model-agnostic approach that attributes each feature’s contribution to a given model prediction, based on its marginal effect across all possible feature combinations [65]. In our application, SHAP values reflect the relative importance of each feature in explaining the variance in predicted counterfactual differences. To maintain interpretability, we limited decision trees to three layers and neural networks to five hidden units per layer.

All analyses were conducted using R (version 4.3.1) [66] and RStudio (version 2023.06.0) [67]. We used the R package *tidymodels* to implement all the machine learning models [68]. The R code used for analytical cohort construction, model development, and data visualization is publicly available at https://github.com/kehezhang12/MOUD_Disparity_DigitalTwins.

## Results

### Patient and episodes characteristics

**Table 1** summarizes the demographic, clinical, and treatment episode characteristics of the analytical cohort. Most of the patients were male (60.8%), residing in the South (34.2%), aged 25–34 years (33.7%) and were covered under commercial insurance (76.0%). Approximately 7.2% had a history of alcohol disorder, and 31.2% reported non-opioid drug use 12 months preceding the first buprenorphine prescription. Mental health conditions were prevalent, with anxiety (22.6%) and depression (22.3%) being the most common diagnoses. Outpatient services were utilized by 90.6% of patients, while 19.4% accessed psychiatric services. Patients had an average of 2.3 treatment episodes, with a median duration of 63 days. Most episodes are discontinued with buprenorphine treatment (N = 215,921; 71.1%), while only 28.9% episodes lasted 180 days or longer. The average 3-month PDC across all episodes is 73%, with continued episodes having averaged 3-month PDC greater than 90%, compared to 65% for discontinued episodes. Discontinuation was significantly more common among patients aged 18–24 (13.3% vs. 8.3%), those residing in the West (26.9% vs. 18.1%), and those with alcohol use disorder (7.0% vs. 4.7%), non-opioid drug use (29.5% vs. 23.3%), or any mental health diagnosis within 12 months before the index date (all comparisons p < 0.001, chi-square tests).

**Table 1:** Baseline patient demographic and clinical characteristics at patient-level and episode-level.

	Episode-level			Individual-level (N = 131,169)
	Discontinued (N = 215,921)	Continued (N = 87,607)	Overall (N = 303,528)	
**Sex**				
Female	82,335 (38.1%)	32,592 (37.2%)	114,927 (37.9%)	51,426 (39.2%)
Male	133,586 (61.9%)	55,015 (62.8%)	188,601 (62.1%)	79,743 (60.8%)
**Age group**				
18-24	28,820 (13.3%)	7,315 (8.3%)	36,135 (11.9%)	18,409 (14.0%)
25-34	69,623 (32.2%)	28,514 (32.5%)	98,137 (32.3%)	44,174 (33.7%)
35-44	52,218 (24.2%)	24,312 (27.8%)	76,530 (25.2%)	32,054 (24.4%)
45-54	36,296 (16.8%)	15,585 (17.8%)	51,881 (17.1%)	20,701 (15.8%)
55-64	22,953 (10.6%)	9,378 (10.7%)	32,331 (10.7%)	12,357 (9.4%)
65-85	6,011 (2.8%)	2,503 (2.9%)	8,514 (2.8%)	3,474 (2.6%)
**Insurance type**				
Medicaid	24,463 (11.3%)	9,588 (10.9%)	34,051 (11.2%)	19,277 (14.7%)
Medicare	15,330 (7.1%)	8,959 (10.2%)	24,289 (8.0%)	11,973 (9.1%)
Commercial	175,872 (81.5%)	68,956 (78.7%)	244,828 (80.7%)	99,735 (76.0%)
Self-insured	256 (0.1%)	104 (0.1%)	360 (0.1%)	184 (0.1%)
**Region of residence**				
Northeast	38,522 (17.8%)	16,666 (19.0%)	55,188 (18.2%)	22,388 (17.1%)
Midwest	49,310 (22.8%)	22,456 (25.6%)	71,766 (23.6%)	35,610 (27.1%)
South	70,034 (32.4%)	32,639 (37.3%)	102,673 (33.8%)	44,882 (34.2%)
West	58,055 (26.9%)	15,846 (18.1%)	73,901 (24.3%)	28,289 (21.6%)
**Alcohol disorder**				
No	200,718 (93.0%)	83,458 (95.3%)	284,176 (93.6%)	121,771 (92.8%)
Yes	15,203 (7.0%)	4,149 (4.7%)	19,352 (6.4%)	9,398 (7.2%)
**Non-opioid drug use**				
No	152,122 (70.5%)	67,179 (76.7%)	219,301 (72.3%)	90,215 (68.8%)
Yes	63,799 (29.5%)	20,428 (23.3%)	84,227 (27.7%)	40,954 (31.2%)
**Schizophrenia**				
No	214,407 (99.3%)	87,236 (99.6%)	301,643 (99.4%)	130,130 (99.2%)
Yes	1,514 (0.7%)	371 (0.4%)	1,885 (0.6%)	1,039 (0.8%)
**Bipolar disorder**				
No	203,693 (94.3%)	84,258 (96.2%)	287,951 (94.9%)	123,745 (94.3%)
Yes	12,228 (5.7%)	3,349 (3.8%)	15,577 (5.1%)	7,424 (5.7%)
**Anxiety**				
No	167,622 (77.6%)	72,625 (82.9%)	240,247 (79.2%)	101,581 (77.4%)
Yes	48,299 (22.4%)	14,982 (17.1%)	63,281 (20.8%)	29,588 (22.6%)
**Post-traumatic stress disorder**				
No	209,884 (97.2%)	85,837 (98.0%)	295,721 (97.4%)	127,079 (96.9%)
Yes	6,037 (2.8%)	1,770 (2.0%)	7,807 (2.6%)	4,090 (3.1%)
**Depressive disorder**				
No	166,752 (77.2%)	72,437 (82.7%)	239,189 (78.8%)	101,863 (77.7%)
Yes	49,169 (22.8%)	15,170 (17.3%)	64,339 (21.2%)	29,306 (22.3%)
**Outpatient visits**				
No	19,112 (8.9%)	8,364 (9.5%)	27,476 (9.1%)	12,387 (9.4%)
Yes	196,809 (91.1%)	79,243 (90.5%)	276,052 (90.9%)	118,782 (90.6%)
**Psychiatric visits**				
No	164,204 (76.0%)	70,048 (80.0%)	234,252 (77.2%)	105,697 (80.6%)
Yes	51,717 (24.0%)	17,559 (20.0%)	69,276 (22.8%)	25,472 (19.4%)
**Medication-assisted treatment**				
No	215,917 (100.0%)	87,606 (100.0%)	303,523 (100.0%)	131,166 (100.0%)
Yes	4 (0.0%)	1 (0.0%)	5 (0.0%)	3 (0.0%)
**Telehealth visits**				
No	212,541 (98.4%)	85,557 (97.7%)	298,098 (98.2%)	128,108 (97.7%)
Yes	3,380 (1.6%)	2,050 (2.3%)	5,430 (1.8%)	3,061 (2.3%)
**Induction visit**				
No	211,479 (97.9%)	86,282 (98.5%)	297,761 (98.1%)	129,026 (98.4%)
Yes	4,442 (2.1%)	1,325 (1.5%)	5,767 (1.9%)	2,143 (1.6%)
**Buprenorphine treatment**				
No	214,566 (99.4%)	86,925 (99.2%)	301,491 (99.3%)	129,981 (99.1%)
Yes	1,355 (0.6%)	682 (0.8%)	2,037 (0.7%)	1,188 (0.9%)
**Social Vulnerability Index**				
Low	24,013 (11.1%)	12,749 (14.6%)	36,762 (12.1%)	17,506 (13.3%)
Low-Medium	62,960 (29.2%)	30,480 (34.8%)	93,440 (30.8%)	42,281 (32.2%)
Medium-High	62,135 (28.8%)	24,729 (28.2%)	86,864 (28.6%)	38,118 (29.1%)
High	66,813 (30.9%)	19,649 (22.4%)	86,462 (28.5%)	33,264 (25.4%)
**Episode duration (days)**				
Mean (SD)	51.1 (43.7)	588 (512)	206 (369)	–
Median [Min, Max]	30.0 [1.00, 179]	419 [180, 7,010]	63.0 [1.00, 7,010]	–
**3-month PDC** ^*^				
Mean (SD)	0.65 (0.33)	0.92 (0.21)	0.73 (0.32)	0.73 (0.34)
Median [Min, Max]	0.70 [0.01, 1.00]	1.00 [0.01, 1.00]	0.86 [0.01, 1.00]	1.00 [0.01, 1.00]

Abbreviations: SD – standard deviation; Min – minimum; Max- maximum.

* The 3-month PDC is defined at episode level to assess adherence over a 90-day period by calculating the proportion of the 90-day interval that is covered by the episode duration.

### First-stage Virtual Twins: Classification model

**Table 2** presents the predictive performance of different machine learning models on the testing dataset, including C-statistics and AUC-PR. The initial evaluation with the baseline predictors achieved the following C-statistics: logistic regression (0.595), decision tree (0.567), random forest (0.608), gradient boosting machine (0.604), and neural network (0.605). Performance improved with the inclusion of contextual SVI, particularly for random forest (0.632) and gradient boosting tree (0.643), suggesting the importance of contextual SDOH in predicting discontinuation risk. Substantial improvements were observed after including the 3-month PDC, known to be highly predictive of treatment adherence. With PDC, C-statistics increased significantly: logistic regression (0.767), decision tree (0.733), random forest (0.785), GBM (0.789), and neural network (0.773). ROC and Precision-recall curves are shown in the Figure A in S1 Appendix, and additional metrics including accuracy, precision, recall and F1 score are available in Tables E-G in S1 Appendix. GBM demonstrated the best performance in Models 2 and 3 and was selected for estimating counterfactual probabilities for Stage-two analysis.

**Table 2:** Model predictive performance from Stage-one classification model by different machine learning algorithms.

Algorithms	Model 1		Model 2		Model 3	
	AUC-ROC	AUC-PR	AUC-ROC	AUC-PR	AUC-ROC	AUC-PR
Logistic regression	0.595	0.360	0.608	0.372	0.767	0.527
Random Forest	0.608	0.375	0.632	0.398	0.785	0.559
Gradient Boosted Machine	0.604	0.371	0.643	0.408	0.789	0.572
Decision Tree	0.567	0.539	0.577	0.520	0.733	0.677
Neural Network	0.605	0.370	0.616	0.379	0.773	0.535

**Fig 3:**
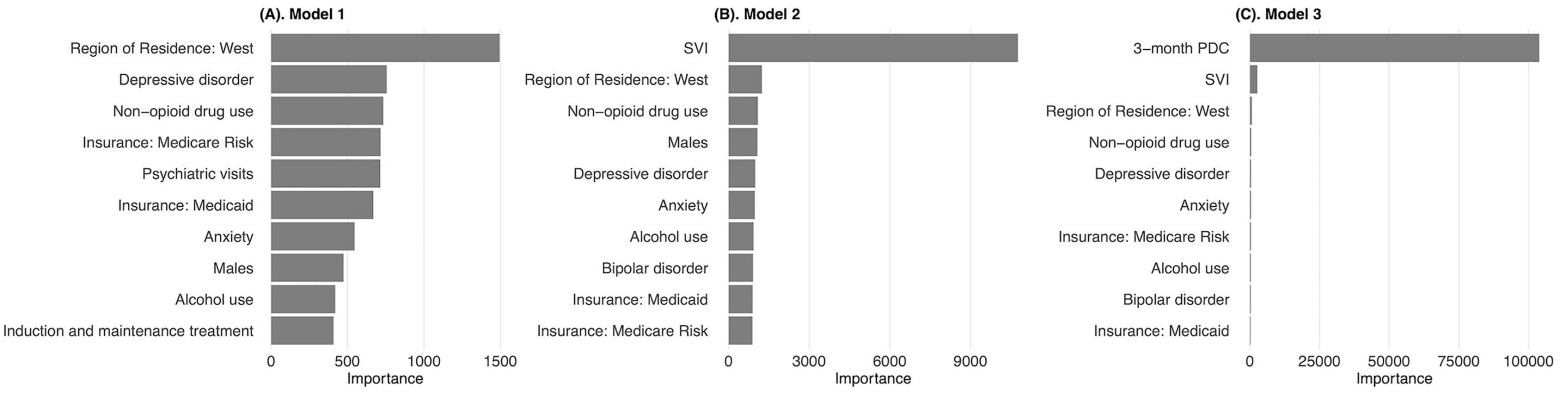
Variable importance plots from the random forest models for Stage-one classification model.

Variable importance plots from the random forest models across all three settings are displayed in **Fig 3**. When using only baseline characteristics, region of residence was the most influential predictor, followed by depression disorder and non-opioid drug use. After incorporating SVI, it became the top predictor, confirming that community-level SDOH factors significantly affect treatment duration. However, with the addition of 3-month PDC, SVI’s importance reduced substantially, as PDC accounted for a large proportion of the variance in treatment discontinuation in this cohort.

### Second-stage Virtual Twins: Regression model

**Fig 4:**
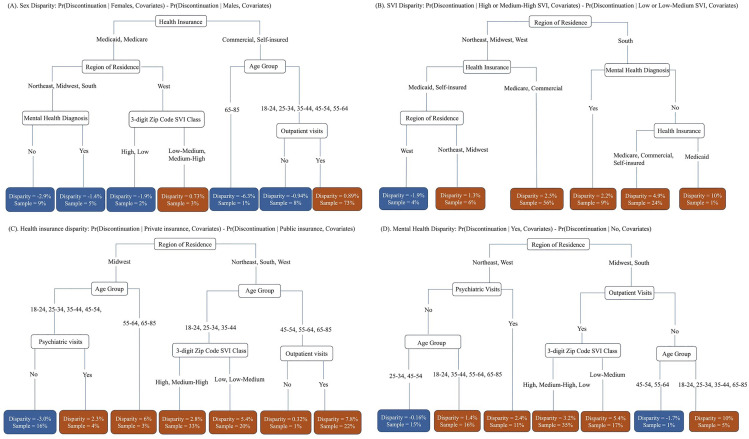
Decision tree diagram from the Stage-two regression model for different types of disparity: **(A)**. **Sex Disparity; (B). SVI Disparity; (C). Health Insurance Disparity (D). Mental Health Disparity.**

For the second-stage analysis, we examined six types of disparities that have been documented in prior research: sex, age, SVI, health insurance, geographic region, and mental health diagnosis. The decision tree for sex disparity (**Fig 4A**) showed overall minimum differences between females and males, with the largest observed among older adults (aged 65–85) with private insurance (commercial or self-insured). Females in this subgroup had a 6.3% lower probability of treatment discontinuation (1% of the episodes; N = 3035). The SVI disparity tree (**Fig 4B**) showed greater discontinuation risks for patients from high-SVI areas, particularly those living in the South without recent mental health diagnoses. Specifically, discontinuation probability increased by 10% for Medicaid patients (1% of the episodes) and by 4.9% for patients with other insurance (24% of the episodes) in this subgroup. Patients with private insurance (commercial or self-insured) generally had higher risks of discontinuation than those with public insurance (**Fig 4C**). Among adults residing in the South, West or Northeast aged 45–85 with recent outpatient visits, having private insurance increased discontinuation risk by 7.8% (22% of the episodes). Mental health diagnosis also contributed to substantial disparities (**Fig 4D**) but varied geographically. In the Midwest and South regions, patients aged 18–44 or 65–85 with no recent outpatient visits, having mental health diagnosis increased the risk of discontinuation by 10% (5% of the episodes). Even among patients with recent outpatient visits, mental health diagnosis increased the risk by 5.4% for those living in low-to-medium SVI areas from Midwest or South (17% of the episodes).

**Fig 5:**
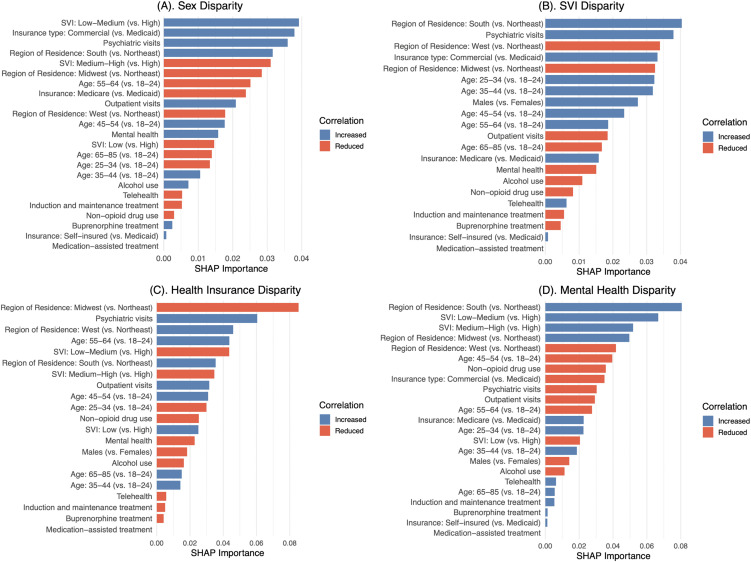
SHAP variable importance plots from the Stage-two neural network regression model: **(A)**. **Sex Disparity; (B). SVI Disparity; (C). Health Insurance Disparity (D). Mental Health Disparity.**

To validate the decision tree findings, we then examined variable importance using SHAP values derived from neural network regression models, which quantifies how much each predictor contributes to the estimated disparity in treatment discontinuation risk between groups. Positive SHAP values indicate predictors associated with increased disparity, that is, a larger difference in discontinuation risk between the comparison groups, while negative values indicate reduced disparity. As shown in **Fig 5**, SVI and region of residence consistently ranked among the most influential contributors to disparities. Insurance type, psychiatric service utilization, and age are also key predictors, highlighting the role of both sociodemographic and behavioral health factors in shaping subgroup differences in treatment duration.

Results for age and geographic region disparities are provided in Figures B and C in S1 Appendix. Overall, patients aged 35–49 had lower discontinuation risk compared to younger (18–34) and older adults (50–85). Additionally, patients in the Western region, particularly older adults (65–85) covered by Medicare or commercial insurance and living in areas with high social vulnerability, experienced notably higher discontinuation risks.

## Discussion

In this nationwide, longitudinal cohort study of OUD patients, approximately 57% treatment episodes were discontinued within 90 days of buprenorphine initiation, and 71% discontinued by 180 days, resulting in a retention rate of only 29%. These findings highlight the challenges of retaining patients in buprenorphine treatment. The Stage-one analysis identified the 3-month PDC as the strongest predictor of treatment discontinuation, underscoring the importance of medication adherence in early treatment stages to promote long-term treatment retention. These findings align closely with previous studies by Faysal et al. (2024) [45] and Hasan et al. (2021) [46], suggesting that interventions focused on improving adherence during the initial treatment phase may be beneficial to improve the overall retention rate.

Our second-stage analysis revealed significant disparities in buprenorphine discontinuation related to SVI, insurance type, region of residence, mental health status, and age. Although prior studies have identified gender-based disparities in OUD treatment retention, citing barriers faced by women such as caregiving responsibilities, financial instability, and limited access to gender-responsive services [69,70], our analysis showed minimum differences in discontinuation risk between females and males. In contrast, disparities related to social vulnerability were more pronounced, especially among patients in high-SVI areas in the South, enrolled in Medicaid, and without a mental health diagnosis. Yet, SVI disparities were mitigated among Southern patients with mental health diagnoses, potentially reflecting the protective effects of psychiatric services. These findings suggest structural barriers, such as inadequate healthcare infrastructure, may disproportionately affect vulnerable populations. Federally Qualified Health Centers, primarily located in underserved communities, could play a key role in addressing these disparities by offering integrated primary care, behavioral health, and substance use services [71].

Mental health diagnosis itself was associated with higher discontinuation risk, especially among younger (18–44) and older (65–85) adults in the Midwest or South without recent outpatient visits. This aligns with previous research showing lower adherence among patients with psychiatric conditions [72–74], possibly reflecting prioritization of mental health treatment over substance use treatment [75]. These findings indicate an urgent need for integrated care models to concurrently address mental health and substance use disorders.

Regarding insurance type, patients with public insurance had lower discontinuation risk overall. This may reflect broader and more stable coverage in Medicaid for both immediate- and extended- release buprenorphine, with minimal prior authorization and quantity limits [76]. Yet, privately insured individuals, especially those aged 45 and older with outpatient visits in the West, South, or Northeast, had a significantly higher discontinuation risk (7.8%). This difference may be attributed to higher cost-sharing and more frequent utilization management in private plans, which can delay or interrupt treatment [77]. In addition, Medicaid expansion has been associated with increased receipt of medications for OUD in outpatient settings [78], and removal of prior authorization requirements has been linked to increased buprenorphine prescribing in Medicaid populations [79], underscoring the role of administrative barriers in treatment retention. Among publicly insured individuals, younger patients (18–54) in the Midwest who lacked psychiatric service utilization showed elevated discontinuation risks, highlighting the potential protective effects of psychiatric services. Integrating behavioral health care, particularly for younger publicly insured patients, may significantly enhance treatment continuity, consistent with evidence supporting the benefits of concurrent behavioral therapies [80]. Tailored approaches that address psychiatric care access and insurance-related barriers are critical for MOUD treatment retention.

We observed age-related disparities in buprenorphine treatment discontinuation. Patients aged 35–49 years showed a lower risk of discontinuation compared to rest of the adult population (those aged 18–34 and 50 + combined). Previous studies similarly report age gradients in buprenorphine retention, with younger adults (18–49) generally exhibiting lower retention compared with those aged ≥50, and a pattern suggesting decreasing retention with decreasing age [81,82]. Although our findings are directionally consistent with this literature, direct comparison remains challenging given the heterogeneous and nonstandardized age categorizations used across studies. We were particularly interested in the 35–49 age group because national surveillance data indicate that adults in this age range account for a substantial proportion of individuals actively receiving OUD treatment [16]. Considered alongside our finding of lower discontinuation risk in this group, their greater representation in treatment may reflect stronger engagement in care, which could contribute to the lower discontinuation risk observed in our analysis.

In addition, we also observed notable regional differences. Individuals in the Western U.S., especially older adults enrolled in Medicare or commercial insurance in high-SVI areas, had the highest discontinuation risks. These findings may reflect geographic disparities in provider availability, restrictive Medicaid policies, and infrastructure barriers such as limited access to waivered prescribers and longer travel distances in rural or socially vulnerable regions [83]. Tailored strategies addressing regional healthcare access gaps and age-specific needs are essential to improve buprenorphine treatment retention.

Our study has several strengths. First, we integrate a geographically referenced contextual measure of social vulnerability, which provided insights beyond individual-level predictors and significantly improved the predictive accuracy. Second, unlike previous studies that were limited by data coverage and demographics, our study utilizes a nationwide, longitudinal cohort, providing more generalizable findings. Third, our use of episode-level data, rather than patient-level data, enables a more granular capture of treatment episode discontinuation. Fourth, our application of a two-stage Virtual Twins analytical framework enables identification of vulnerable subgroups and quantification of disparities attributable to specific risk factors. This approach, while previously applied in substance abuse research [49,50], is the first in MOUD treatment retention, addressing the limitations of single prediction models that fail to capture heterogeneous effects across diverse populations [45,46].

Despite the strengths, we should also acknowledge the limitations. First, our demographic data lacked patient race and ethnicity, a key disparity reported by many other studies [52,84]. Second, due to the limitation of the dataset, we could not account for concurrent medications taken by patients [45] or their initial amount of dosage, despite the evidence that a low initial dose of buprenorphine (4mg or less) was seen as the strongest risk factor driving discontinuation [17]. In addition, although all major buprenorphine formulations were included, long-acting injectable products accounted for only 0.35% of prescriptions, which precluded meaningful comparisons between short- and long-acting formulations. Future studies with greater uptake of extended-release formulations are needed to evaluate formulation-specific retention patterns. Third, geographic location was derived from each patient’s most recent enrollment record and treated as time-invariant. Therefore, we were unable to capture residential changes over time, which may introduce some misclassification if patients relocated during the study period. Finally, although the use of the Virtual Twins framework is a novel approach to quantifying OUD disparities, it currently lacks uncertainty assessment, which warrants investigation in future studies.

## Conclusion

Leveraging a nationwide longitudinal cohort and a two-stage Virtual Twins model, our study identified and quantified vulnerable subgroups with pronounced disparities in sex, age, insurance, region of residence, mental health status, and SVI. Our key innovation is the incorporation of contextual SDOH, allowing us to capture key risk and protective factors and providing actionable insights for tailored policy interventions. This study has shown that different disparities exist and persist in MOUD treatment retention, and psychiatric service utilization consistently emerged as protective against treatment discontinuation across various disparities. These findings highlight the importance of targeted interventions, including enhanced psychiatric care, region-specific programs, and strengthened community support, to address structural barriers and improve equitable access and retention in medication for OUD treatment.

## Supporting information

S1 Appendix Supplemental materials including supplemental figures, tables, and method descriptions. **Table A**. List of variables defined at patient, episode and community level. Each variable may be coded differently for Stage-one and Stage-two analysis. **Table B.** List of Diagnosis and Healthcare utilization. **Table C.** Healthcare utilization identified using CPT/HCPCS codes. **Table D.** List of variables included in each model. **Supplemental Methods:** Stage-one Model Training and Parameter Tuning. **Figure A.** AUC-ROC and AUC-PR Curve for Stage-one digital twin analysis: (A). Model 1; (B). Model 2; (C). Model 3. **Table E.** Results from Stage-one model 1: baseline predictors only. **Table F.** Results from Stage-one model 2: baseline predictors + SVI. **Table G.** Results from Stage-one model 3: baseline predictors + SVI + PDC. **Figure B.** Decision tree results for (A). Geographic region disparity and (B). Age disparity. **Figure C.** SHAP importance from neural network model for (A). Geographic region disparity and (B). Age disparity. **Supplemental Results:** Sensitivity Analysis on Gaps of Supply, Sensitivity Analysis on PDC 30 vs. PDC 90.
(DOCX)
