# Supplementary material for: Subgroup identification of disparities in buprenorphine discontinuation in opioid-use disorder: A Virtual Twins machine learning approach using nationwide United States claims data, 2006–2022
Source: PLOS Ment Health. 2026 Apr 9;3(4):e0000469. doi: 10.1371/journal.pmen.0000469 (PMC13065073; doi:10.1371/journal.pmen.0000469)
Supplement: S1 Appendix — Table A. List of variables defined at patient, episode and community level. Each variable may be coded differently for Stage-one and Stage-two analysis. Table B. List of Diagnosis and Healthcare utilization. Table C. Healthcare utilization identified using CPT/HCPCS codes. Table D. List of variables included in each model. Supplemental Methods: Stage-one Model Training and Parameter Tuning. Figure A. AUC-ROC and AUC-PR Curve for Stage-one digital twin analysis: (A). Model 1; (B). Model 2; (C). Model 3. Table E. Results from Stage-one model 1: baseline predictors only. Table F. Results from Stage-one model 2: baseline predictors + SVI. Table G. Results from Stage-one model 3: baseline predictors + SVI + PDC. Figure B. Decision tree results for (A). Geographic region disparity and (B). Age disparity. Figure C. SHAP importance from neural network model for (A). Geographic region disparity and (B). Age disparity. Supplemental Results: Sensitivity Analysis on Gaps of Supply, Sensitivity Analysis on PDC 30 vs. PDC 90. (DOCX) [file pmen.0000469.s001.docx]

**Supplemental Materials**

**Subgroup identification of disparities in buprenorphine discontinuation in opioid-use disorder: A Virtual Twins machine learning approach using nationwide United States claims data, 2006-2022**

**Table A**. List of variables defined at patient, episode and community level. Each variable may be coded differently for Stage-one and Stage-two analysis.

| Level | Variables | Stage 1 & 2 as predictors | Stage 2 as Outcome (Binary) |
| --- | --- | --- | --- |
| Patient | Age | 18-24; 25-34; 35-44; 45-54; 55-64; 65-85 | 18-34 and 50-85; 35-49 |
|  | Sex | Females; Males | Females; Males |
|  | Insurance type | Medicaid; Medicare; Commercial; Self-insured | Public; Private |
|  | Region of residence | West; East; Midwest; South | West; Other regions |
|  | Diagnosis (Table B) | Yes; No | Yes; No |
|  | Procedures (Table C) | Yes; No | Yes; No |
|  | 3-month PDC | Proportion of days covered in the first 3 months after buprenorphine treatment initiation | - |
| Episode | Episode discontinuation | Yes ($<$180 days) or No ($\geq$ 180 days) | - |
| Community | Social vulnerability index | Continuous scale ranging from 0 to 1 | High and Medium-High;  Low-Medium and Low |

**Table B.** List of diagnostic conditions with ICD-9 and ICD-10 code definitions.

| **Conditions** | **ICD-10 codes** | **ICD-9 codes** |
| --- | --- | --- |
| Alcohol use disorder | F10 | 305.00, 305.01, 305.02, 303.xx |
| Non-opioid drug use disorder | F12–F19 | 292.0x, 292.2, 292.11, 292.81, 292.82, 292.84, 292.89, 292.9, 294.8x, 304.10, 304.11, 304.20, 304.21, 304.30, 304.31, 304.33, 304.40, 304.43, 304.50, 304.53, 304.60, 304.63, 304.90, 304.93, 305.1, 305.20, 305.21, 305.30, 305.33, 305.40, 305.43, 305.60, 305.61, 305.70, 305.71, 305.80, 305.81, 305.90, 305.93 |
| Schizophrenia | F20–F21, F25 | 295.30, 295.10, 295.90, 295.60, 295.20, 295.40, 295.80, 301.22, 295.70 |
| Bipolar disorder | F31, F34.0 | 296.4, 296.5, 296.6, 296.7, 296.80, 296.89 |
| HIV/AIDS | B20-B24 | 042, 043, 044 |
| Depressive disorder | F32, F33, F34.1 | 296.2, 296.3, 296.82, 300.4, 301.12, 311 |
| Anxiety | F40-F42 | 300.2, 300.0, 300.3, 306.3 |
| Post-traumatic stress disorder (PTSD) | F43.1 | 309.81 |

**Table C.** Healthcare utilization measures defined by CPT/HCPCS codes.

| **Category** | **CPT/HCPCS Codes** | **Description** |
| --- | --- | --- |
| Buprenorphine counseling | G2080 | An additional 30 minutes of counseling per week for medication-assisted treatment (MAT) |
| Buprenorphine treatment services | T1015 | Medicaid code for clinic visits related to opioid use disorder treatment services |
| Induction and maintenance services | 99354-99355 | Prolonged services beyond the typical office visit time for induction or maintenance treatment. |
| Outpatient medical services | 99201-99205, 99211-99215, 99241-99245 | Office or outpatient visits to physicians related to medical treatment |
| Outpatient psychiatric services | 90801, 90802, 90804-90815, 90845-90857, 90862-90899 | Psychiatric evaluations and therapy services related to substance use disorders |
| Telehealth visits | 99441-99443 | Telephone evaluation and management services, which may include counseling or MAT. |

**Table D.** List of variables included in each model in Stage-one analysis.

| **Model** | **Variables** |
| --- | --- |
| Model1 | Sex, patient region of residence, age group, insurance type, diagnosis of schizophrenia, bipolar, anxiety, PTSD, depressive disorder, alcohol use disorder; outpatients visits, psychiatric health service utilization, telehealth service, induction and maintenance treatment, buprenorphine treatment service |
| Model2 | All variables in Model 1 + Rescaled SVI score (0-1) |
| Model3 | All variables in Model 2 + 3-month PDC (Proportion of days covered) |

*Note: the 3-month PDC is defined at patient level to assess adherence over a 90-day period by calculating the proportion of the 90-day interval that is covered by the episode duration.*

**Supplemental Methods**

***Stage-one Model Training and Parameter Tuning***

For the stage-one classification model, we trained and compared five machine learning algorithms: logistic regression, decision tree, random forest, gradient boosted machine (GBM), and neural network. The dataset was split into 80% training and 20% testing sets. We performed hyperparameter tuning using grid search within cross-validation. For random forest, we tuned the number of trees (fixed at 500), number of randomly selected predictors (4, 5, 6, 7), and minimum node size (2, 8, 14, 20). GBM models were tuned for number of trees (200, 400, 600), tree depth (10, 12, 14), and learning rate (0.1, 0.01, 0.001). For decision trees, we adjusted tree depth (1–6) and cost-complexity parameters (0.1, 0.01, 0.001, 0.0001). Neural networks were tuned for the number of hidden units (4, 8, 12, 16) and regularization strength (0.1, 0.01, 0.001, 0.0001).

**Figure A.** AUC-ROC and AUC-PR Curve for Stage-one digital twin analysis: (A). Model 1; (B). Model 2; (C). Model 3.

**Table E.** Results from Stage-one model 1: baseline predictors only

| **Model** | **AUC-ROC** | **AUC-PR** | **Precision** | **Recall** | **Accuracy** | **F1** |
| --- | --- | --- | --- | --- | --- | --- |
| Logistic regression | 0.595 | 0.360 | 0.341 | 0.641 | 0.539 | 0.445 |
| Random Forest | 0.608 | 0.375 | 0.350 | 0.627 | 0.556 | 0.449 |
| Gradient Boosted machine | 0.604 | 0.371 | 0.349 | 0.627 | 0.555 | 0.448 |
| Decision Tree | 0.567 | 0.539 | 0.334 | 0.637 | 0.529 | 0.438 |
| Neural Network | 0.605 | 0.370 | 0.347 | 0.633 | 0.551 | 0.449 |

**Table F.** Results from Stage-one model 2: baseline predictors + SVI

| **Model** | **AUC-ROC** | **AUC-PR** | **Precision** | **Recall** | **Accuracy** | **F1** |
| --- | --- | --- | --- | --- | --- | --- |
| Logistic regression | 0.608 | 0.372 | 0.351 | 0.621 | 0.559 | 0.448 |
| Random Forest | 0.632 | 0.398 | 0.369 | 0.611 | 0.586 | 0.460 |
| Gradient Boosted Machine | 0.643 | 0.408 | 0.402 | 0.505 | 0.640 | 0.448 |
| Decision Tree | 0.577 | 0.520 | 0.347 | 0.566 | 0.568 | 0.430 |
| Neural Network | 0.616 | 0.379 | 0.352 | 0.631 | 0.559 | 0.452 |

**Table G.** Results from Stage-one model 3: baseline predictors + SVI + PDC

| **Model** | **AUC-ROC** | **AUC-PR** | **Precision** | **Recall** | **Accuracy** | **F1** |
| --- | --- | --- | --- | --- | --- | --- |
| Logistic regression | 0.767 | 0.527 | 0.457 | 0.835 | 0.667 | 0.591 |
| Random Forest | 0.785 | 0.559 | 0.508 | 0.779 | 0.719 | 0.615 |
| Gradient Boosted Machine | 0.789 | 0.572 | 0.532 | 0.717 | 0.736 | 0.611 |
| Decision Tree | 0.733 | 0.677 | 0.487 | 0.814 | 0.699 | 0.610 |
| Neural Network | 0.773 | 0.535 | 0.485 | 0.807 | 0.697 | 0.606 |

**Figure B.** Decision tree results for (A). Geographic region disparity and (B). Age disparity.

**Figure C.** SHAP importance from neural network model for (A). Geographic region disparity and (B). Age disparity.

**Supplemental Results**

***Sensitivity Analysis on Gaps of Supply***

1. 7-day gap in supply of buprenorphine


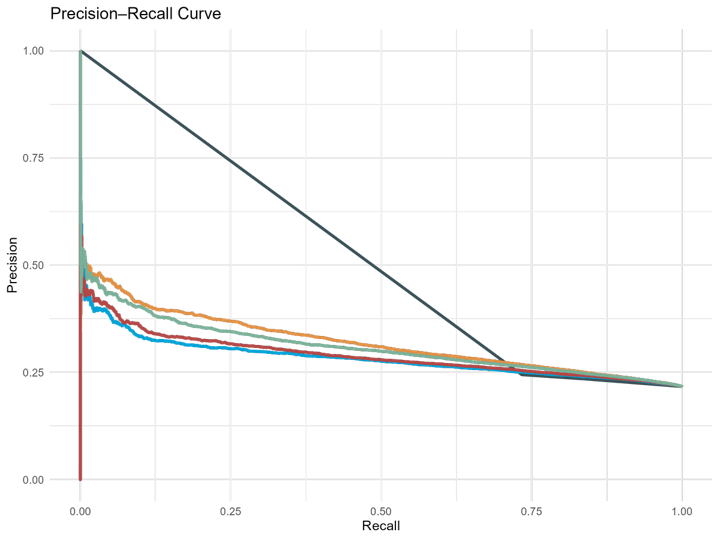

1. 21-day gap in supply of buprenorphine


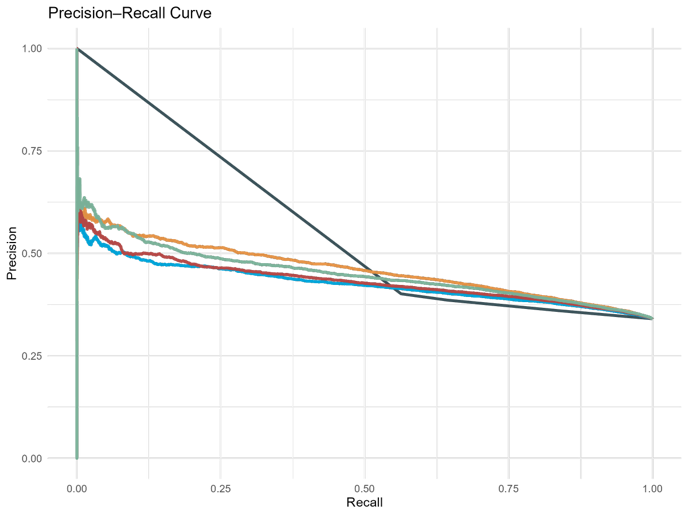

1. 30-day gap in supply of buprenorphine


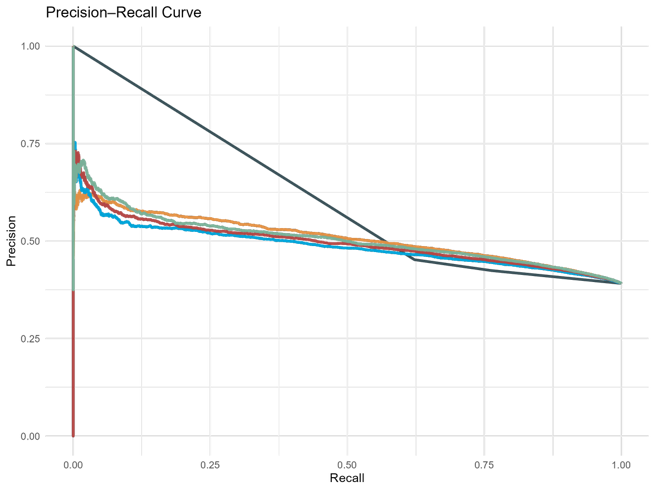

1. 60-day gap in supply of buprenorphine


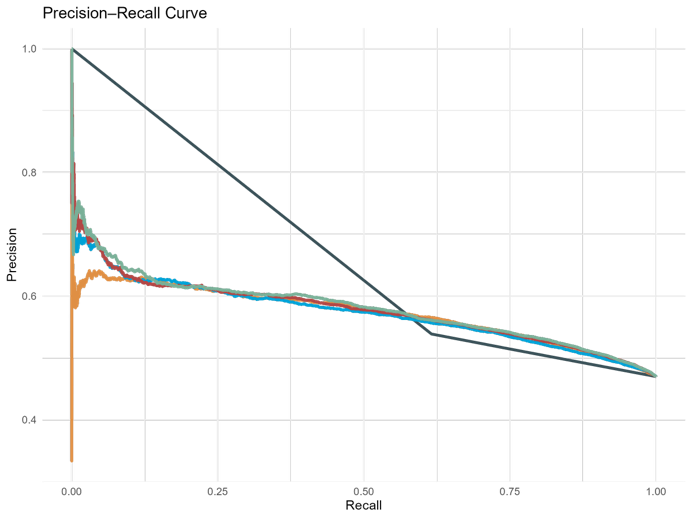

***Sensitivity Analysis on PDC 30 vs. PDC 90***
